# Development of magnetic anionic liposome/atelocollagen complexes for efficient magnetic drug targeting

**DOI:** 10.1080/10717544.2017.1402219

**Published:** 2017-11-15

**Authors:** Yusuke Kono, Taketo Nakai, Hitomi Taguchi, Takuya Fujita

**Affiliations:** a Laboratory of Molecular Pharmacokinetics, College of Pharmaceutical Sciences, Ritsumeikan University, Kusatsu, Japan;; b Ritsumeikan-Global Innovation Research Organization, Ritsumeikan University, Kusatsu, Japan;; c Research Center for Drug Discovery and Development, Ritsumeikan University, Kusatsu, Japan

**Keywords:** Magnetic drug targeting, magnetic field, anionic liposomes, atelocollagen, cationic liposomes

## Abstract

Magnetic nanoparticle-incorporated liposomes (magnetic liposomes) are considered a promising site-specific drug delivery carrier vehicle. With regard to their surface charge, magnetic anionic liposomes (Mag-AL) demonstrate little toxicity in comparison with magnetic cationic liposomes (Mag-CL), whereas their cellular association and uptake efficiency are low. In the current study, we constructed complexes of Mag-AL and atelocollagen (ATCOL), which is a biocompatible and minimally immunogenic biomaterial, to improve the cellular uptake properties of Mag-AL *in vitro* and *in vivo*. The cellular association and/or uptake of Mag-AL in RAW264 cells, a murine macrophage-like cell line, under a magnetic field was significantly increased when Mag-AL was complexed with ATCOL, and the highest cellular association was observed with complexes constructed using 5 µg/mL of ATCOL. The complexes showed liposome concentration-dependent and time-dependent cellular association under a magnetic field, and their cellular uptake efficiency was comparable with that of Mag-CL. In addition, Mag-CL showed significant cytotoxicity in a liposome concentration-dependent manner, whereas Mag-AL/ATCOL complexes produced no cytotoxic effect against RAW264 cells. Furthermore, the efficient cellular association of Mag-AL/ATCOL complexes in RAW264 cells was observed even in the presence of serum, and their liver accumulation was significantly increased at a magnetic field-exposed region after intravenous injection in rats. These results indicate that Mag-AL/ATCOL complexes could be a safe and efficient magnetic responsive drug carrier.

## Introduction

Magnetic nanoparticles (MNPs) have been widely used for not only diagnostic and therapeutic purposes, including magnetic resonance imaging (Lee & Hyeon, 2012; Sharifi et al., 2015), hyperthermia (Silva et al., 2011; Quinto et al., 2015), and targeted drug delivery (Kim et al., 2012; Wahajuddin & Arora, 2012; Tietze et al., 2015), but also bioengineering purposes, such as separation of cells and nucleic acids (He et al., 2014), 3-D cell culture (Ghosh et al., 2016), and immunoassays (Yang et al., 2013). MNPs have been increasingly applied for drug delivery because MNP-based drug carriers possess several advantages, such as tissue-selective accumulation and controlled drug release by means of an external magnetic field (Kim et al., 2012; Wahajuddin & Arora, 2012; Tietze et al., 2015). To succeed in exploiting MNPs as drug carriers, efforts have been made to vest MNPs with specific characteristics, including monodispersity, biocompatibility, and stability (Gupta & Gupta, 2005; Hałupka-Bryl et al., 2014; Prabha & Raj, 2016). In particular, incorporation of MNPs into liposomes is a valuable method to realize such functionalization of MNPs because liposomes are monodispersed vesicles composed of biocompatible and biodegradable lipids, possessing a capacity to load a broad variety of drugs, genes, and proteins (Abu Lila & Ishida, 2017).

Various kinds of MNP-incorporated liposomes (magnetic liposomes) have been developed for tissue-selective drug or gene delivery (Tai et al., 2009; Garnier et al., 2012; Clares et al., 2013). A cationic surface charge provides liposomes with several advantages. For example, cationic liposomes have high monodispersity and stability because of their electrostatic repulsion (Bozzuto & Molinari, 2015). In addition, we and other groups have demonstrated that magnetic cationic liposomes (Mag-CL) electrostatically interact with the anionic regions of the cell surface, resulting in efficient cell uptake (Dandamudi & Campbell, 2007; Shido et al., 2010; Kono et al., 2017). However, cationic lipids and cationic polymers, which are major components of cationic liposomes, have been known to exhibit strong toxicity (Lv et al., 2006). However, anionic liposomes have little toxicity, but their cellular uptake efficiency is relatively low (Kurosaki et al., 2014; Kono et al., 2017). Therefore, we anticipate that the development of safe and efficient magnetic liposomes can be achieved by improving the cellular association and uptake efficiency of magnetic anionic liposomes (Mag-AL).

Atelocollagen (ATCOL) is a highly purified type I collagen derived from calf dermis treated with pepsin to eliminate immunogenic telopeptides at both N- and C-terminals (Sano et al., 2003; Hanai et al., 2006). ATCOL has been widely used as a safe biomaterial for medical applications, plastic surgical instruments, and cosmetic supplies, because it has several beneficial features, such as biocompatibility, biodegradability, and minimal immunogenicity (Sano et al., 2003; Hanai et al., 2006). In addition, ATCOL has been applied for gene delivery, since it possesses a cationic charge under physiological conditions, and forms stable complexes with plasmid DNA (pDNA) or small interfering RNA (siRNA) (Sano et al., 2003; Kuroda et al., 2005; Takeshita et al., 2005; Hanai et al., 2006). A number of reports have demonstrated that ATCOL protects pDNA or siRNA from degradation by nucleases, and increases their cell uptake (Sano et al., 2003; Kuroda et al., 2005; Takeshita et al., 2005; Hanai et al., 2006). Based on these reports, we hypothesize that ATCOL has the potential to improve the cellular association and uptake efficiency of Mag-AL without producing cytotoxicity.

In the current study, we constructed Mag-AL/ATCOL complexes, and evaluated their *in vitro* cellular association properties, cytotoxicity, and *in vivo* liver accumulation under an external magnetic field.

## Materials and methods

### Animals and cell lines

All animal experiments were carried out in accordance with the Guide for the Care and Use of Laboratory Animals as adopted and promulgated by the US National Institutes of Health (Bethesda, MD) and Guidelines for Animal Experiments of Ritsumeikan University (Shiga, Japan). The protocol was approved by the Animal Experimentation Committee of Ritsumeikan University (approval number: BKC2016-016). All efforts were made to minimize the suffering of experimental animals. Seven-week-old male Sprague Dawley rats were purchased from SLC, Inc. (Shizuoka, Japan).

RAW264 cells, a murine macrophage-like cell line, and CT-26 cells, a murine colon carcinoma cell line, were obtained from Riken Bioresource Center (Ibaraki, Japan). The cells were maintained in RPMI 1640 medium supplemented using 10% heat-inactivated fetal bovine serum (FBS), penicillin G (100 U/mL), and streptomycin (100 µg/mL) at 37 °C in 5% CO_2_/95% air.

### Preparation of magnetic liposomes

The magnetic liposomes were prepared according to our previous report (Kono et al., 2017). Briefly, 1,2-distearoyl-*sn*-glycero-3-phospho-(1′-*rac*-glycerol) (DSPG) (Avanti Polar Lipids Inc., Alabaster, AL) and cholesterol, or 1,2-dioleoyl-3-trimethylammonium-propane (Avanti Polar Lipids Inc., Alabaster, AL) and cholesterol were mixed in chloroform at a molar ratio of 1:1 for the preparation of Mag-AL or Mag-CL, respectively. To measure the amount of magnetic liposomes *in vitro* and *in vivo*, both magnetic liposomes were labeled using 5 mol% of DiIC 18(3) (Wako Pure Chemical Industries, Ltd., Osaka, Japan). The lipid mixture was dried through evaporation, followed by vacuum desiccation. The resultant lipid film was suspended in a sterile 5% glucose solution containing 0.1 mg/mL iron oxide (II,III) MNPs (Sigma-Aldrich, St. Louis, MO), and hydrated for 30 min at 70 °C under mechanical agitation. For the preparation of doxorubicin (DXR)-loaded Mag-AL, the lipid film was suspended in 300 mM ammonium sulfate (pH 4.0), and hydrated for 30 min at 70 °C under mechanical agitation. The resultant dispersion was sonicated for 15 min in a bath-type sonicator, and then for 3 min in a prove-type sonicator. The particle sizes and *ζ*-potentials of the magnetic liposomes were measured using a Zetasizer Nano ZS instrument (Malvern Instrument, Worcestershire, UK).

DXR was loaded into Mag-AL by the remote-loading method (Un et al., 2012). Briefly, the external phase of Mag-AL was replaced with phosphate-buffered saline (PBS) (pH 8.0) by gel filtration using a Sephadex G-25 column (PD-10, GE Healthcare, Buckinghamshire, UK). Mag-AL was then incubated with DXR in PBS (pH 8.0) at a lipid-to-drug molar ratio of 10:1, and incubated at 60 °C for 1 h. The unencapsulated DXR was removed by gel filtration.

### Construction of Mag-AL/ATCOL complexes

ATCOL was purchased from Koken Co., Ltd. (Tokyo, Japan). Mag-AL/ATCOL complexes were prepared by gently mixing equal volumes of Mag-AL (final concentration at 10 µg lipid/mL) and ATCOL (final concentration at 1–80 µg/mL), followed by incubation for 20 min at 4 °C. For the cell viability assay and the liposome-concentration dependent cellular association experiment, the complexes were constructed by mixing an equal volume of Mag-AL (final concentration at 1–100 µg lipid/mL) and ATCOL (final concentration at 5 µg/mL). For the *in vivo* experiments, Mag-AL (final concentration at 500 µg lipid/mL) was mixed with an equal volume of ATCOL (final concentration at 5 or 10 µg/mL). The particle sizes and *ξ*-potentials of the complexes were measured using a Zetasizer Nano ZS instrument (Malvern Instrument, Worcestershire, UK). The complexes were stored for 24 h at 4 °C or 37 °C, and photographed at predetermined time points. Transmission electron microscope (TEM) image of the complexes was recorded on a TEM (JEM-1400Plus; JEOL Ltd., Tokyo, Japan) with negative staining using 2% phosphotungstic acid solution (pH 7.0).

### Cellular association and/or uptake experiment

RAW264 cells were seeded on 24-well culture plates at a density of 1 × 10^5^ cells/cm^2^, and cultured for 2 d. The cultured cells were placed on a magnetic plate (OZ Biosciences, San Diego, CA), and the culture medium was replaced with Hank’s Balanced Salt Solution (HBSS) with 5 mM glucose containing 1–100 µg lipid/mL of Mag-AL, Mag-AL/ATCOL complexes, or Mag-CL, and the cells were incubated for 30 min at 37 °C. For the evaluation of the effect of FBS, each magnetic liposome sample was pre-incubated with 20% FBS for 30 min at 37 °C before the experiments were carried out. For the evaluation of time-dependent cellular association, 1 µg lipid/mL of Mag-AL/ATCOL, or Mag-CL was added to each well, and incubated for 30–120 min at 4 °C or 37 °C. Then, the cells were washed twice using ice-cold HBSS, and lysed using lysis buffer (0.05% Triton X-100, 2 mM ethylenediaminetetraacetic acid, 0.1 M Tris, pH 7.8). After centrifugation at 10,000×*g* for 10 min at 4 °C, the amount of Mag-AL in the supernatant was quantified by measuring the DiIC 18(3)-derived fluorescence intensity at a wavelength of 490(ex)/565(em) nm using a SH-8100 microplate reader (Corona Electric Co., Ltd., Ibaraki, Japan). The amount of Mag-AL was normalized with respect to the protein content of the cells using a BCA protein assay kit (TaKaRa Bio Inc., Shiga, Japan).

### Cell viability assay

RAW264 cells were seeded in 24-well culture plates at a density of 1 × 10^5^ cells/cm^2^, and cultured for 2 d. The culture medium was replaced with HBSS containing 1–100 µg lipid/mL of Mag-AL, Mag-AL/ATCOL complexes, or Mag-CL. After incubation for 30 min at 37 °C, cell viability was measured using Cell Counting Reagent SF (Nacalai Tesque, Kyoto, Japan) and an Infinite F200 microplate reader (Tecan Japan Co., Ltd., Kanagawa, Japan). The results are expressed as viability (%).

For evaluation of cytotoxicity by DXR-loaded Mag-AL/ATCOL complexes, CT-26 cells were seeded in 24-well culture plates at a density of 1 × 10^5^ cells/cm^2^ and cultured for 1 d. Various concentrations of DXR-loaded Mag-AL or DXR-loaded Mag-AL/ATCOL complexes (10^−2^ to 10 µM as DRX) were added to the wells and the cells incubated for 60 min at 37 °C. Liposomes were then removed and the culture medium was replaced with fresh RPMI 1640 medium. After 2 d, cell viability was measured.

### DXR release experiment

The release of DXR from Mag-AL was measured by the equilibrium dialysis method. Briefly, DXR-loaded Mag-AL or DXR-loaded Mag-AL/ATCOL complexes were loaded into an Xpress Micro Dialyzer (MWCO: 12,000–14,000, Scienova, Jena, Germany). The dialyzer was placed into PBS containing 5% (w/v) bovine serum albumin and incubated for 480 min at 37 °C. The released DXR was detected by measuring the DXR-derived fluorescent intensity at a wavelength of 490 (ex)/590 (em) nm.

### Fluorescence photographs of DXR-loaded Mag-AL/ATCOL complexes associated with RAW264 cells

To visualize the cellular association of Mag-AL and DXR by fluorescence microscopy, Mag-AL was labeled with 1% (N-(fluorescein-5-thiocarbamoyl)-1,2-dihexadecanoyl-*sn*-glycero-3-phosphoethanolamine (Thermo Fisher Scientific K.K., Kanagawa, Japan). DXR-loaded Mag-AL or DXR-loaded Mag-AL/ATCOL complexes were exposed to CT-26 cells seeded on 24-well plates for 60 min at 37 °C with or without a magnet. The cells were then washed twice using ice-cold HBSS and observed by fluorescent microscopy (BZ-X710, Keyence Co., Osaka, Japan).

### *In vivo* hepatic disposition experiment

Rats were anesthetized through an intraperitoneal injection of pentobarbital sodium (20 mg/kg) with inhalation of isoflurane, and a disk-shaped magnet (20 mm diameter) was inserted under the liver. After an intravenous injection of Mag-AL/ATCOL complexes or Mag-CL (2 mg lipid/kg), blood was collected from the jugular vein at predetermined time points, followed by centrifugation at 4000×*g* for 15 min at 4 °C to obtain plasma. Two hours after the injection, the rats were sacrificed and their livers were collected, rinsed using HBSS, and weighed. The extraction of magnetic liposomes from the plasma and liver was carried out according to the method reported by Shiraishi et al. (2013). The liver was homogenized with saline, and plasma or liver homogenate was added with a three-fold excess of chloroform:methanol (3:1). The resultant mixture was centrifuged at 2000×*g* for 15 min at 20 °C, and the amount of Mag-AL in the organic layer was quantified by measuring the DiIC 18(3)-derived fluorescence intensity. The amount of Mag-AL was normalized with respect to the protein content of the cells.

### Statistical analysis

The results are presented as the mean + or ± standard deviation (SD) of more than three experiments. Analysis of variance (ANOVA) was used to test the statistical significance of differences between groups. Two-group comparisons were performed using Student’s *t* test. Multiple comparisons between control groups and other groups were performed using Dunnett’s test, and those between all groups were carried out using a Tukey–Kramer test.

## Results

### Physicochemical properties of Mag-AL/ATCOL complexes

**Figure 1.**
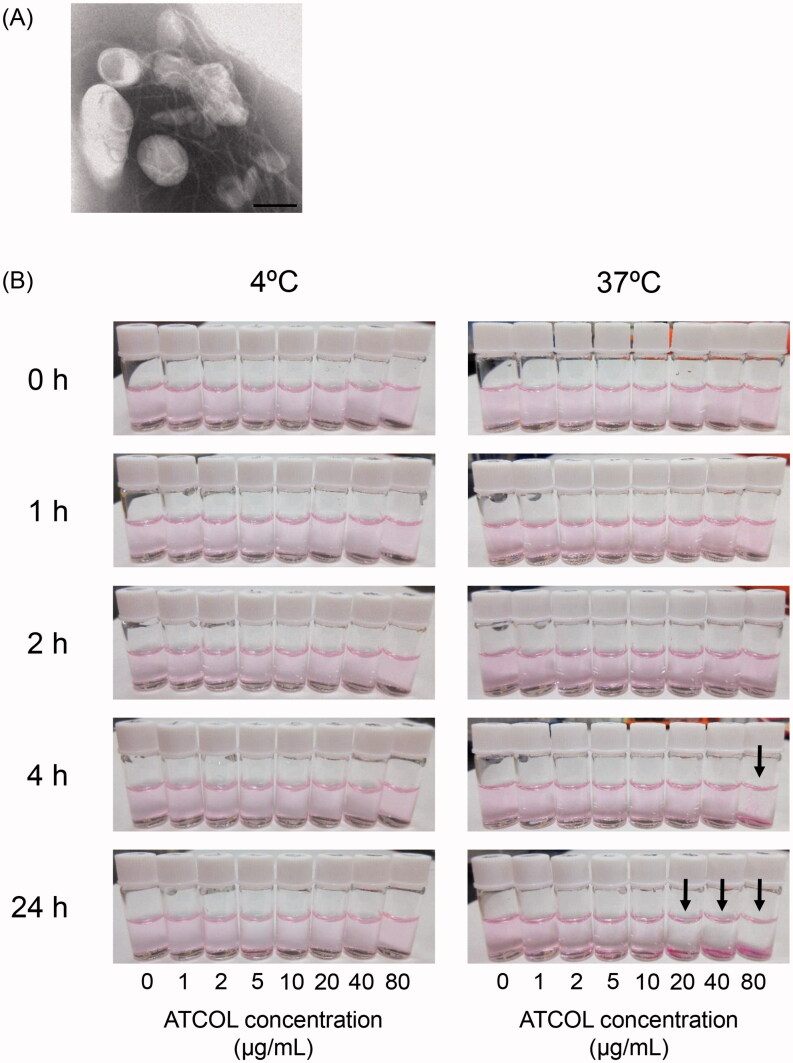
TEM image and photographs of Mag-AL/ATCOL complexes. (A) Magnetic anionic liposomes/atelocollagen (Mag-AL/ATCOL) complexes constructed using 5 µg lipid/mL of ATCOL were observed by TEM with negative staining. Scale bar, 100 nm. (B) Mag-AL/ATCOL complexes were prepared by mixing Mag-AL (final concentration at 10 µg lipid/mL) with various concentrations of ATCOL (final concentration at 1–80 µg/mL). The complexes were stored at 4 °C or 37 °C, and observed at 0, 1, 2, 4, and 24 h after preparation. Black arrows indicate aggregation.

We prepared Mag-AL and constructed complexes with ATCOL via electrostatic interaction. The particle size and *ζ*-potential of the Mag-AL was approximately 120 nm and −55 mV, respectively (Table 1). When Mag-AL was mixed with ATCOL, both particle size and *ζ*-potential gradually increased as the concentration of ATCOL was increased. In addition, the TEM image clearly visualized the structure of Mag-AL/ATCOL complexes, and showed that ATCOL was coated on the surface of Mag-AL (Figure 1(A)). We also confirmed that Mag-AL/ATCOL complexes were uniformly dispersed for 24 h at 4 °C, regardless of the concentration of ATCOL (Figure 1(B)). However, the aggregation of the complexes with a high ATCOL concentration (20–80 µg/mL) was visually confirmed when the complexes were maintained for more than 4 h at 37 °C.

**Table 1.** Particle sizes and *ζ*-potentials of magnetic anionic liposome/atelocollagen (ATCOL) complexes.

ATCOL concentration (μg/mL)	Particle size (nm)	*ζ*-potential (mV)
0	124.3 ± 13.7	−54.6 ± 6.4
1	122.5 ± 4.4	−54.0 ± 5.3
2	132.7 ± 16.4	−50.8 ± 2.1
5	135.1 ± 14.6	−33.1 ± 2.7
10	171.4 ± 15.8	−4.2 ± 1.1
20	275.3 ± 4.0	10.9 ± 1.9
40	441.3 ± 18.9	17.4 ± 1.3
80	528.6 ± 21.2	19.7 ± 2.5

Each value represents the mean ± SD (*n* = 4).

### Effect of ATCOL concentration on the cellular association and cytotoxicity of Mag-AL/ATCOL complexes in RAW264 cells

**Figure 2.**
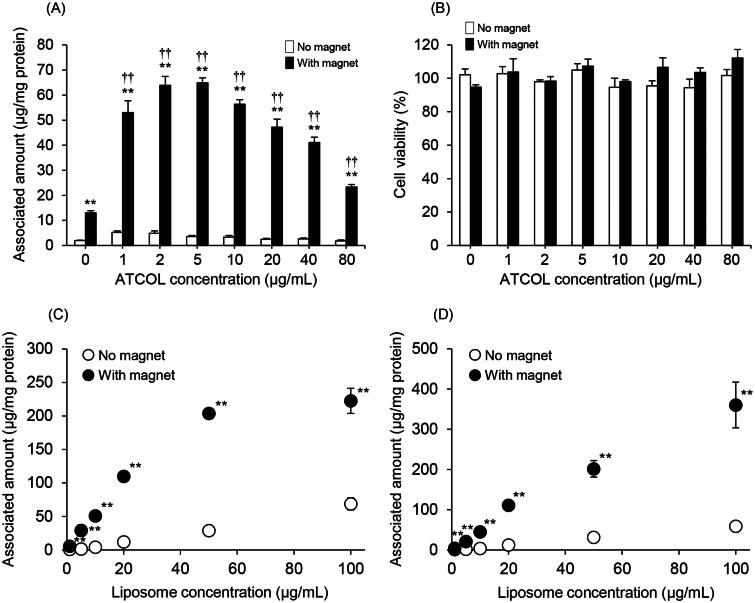
Cellular association and cytotoxicity of Mag-AL/ATCOL complexes in RAW264 cells. Effect of ATCOL concentration on the cellular association and/or uptake of Mag-AL/ATCOL complexes (A) and the cell viability of RAW264 cells (B). A total of 10 µg lipid/mL of the complexes was added to each well, and incubated for 30 min at 37 °C in the presence or absence of a magnetic field. Each value represents the mean + SD (*n* = 4). Liposome concentration-dependent cellular association and/or uptake of Mag-AL/ATCOL complexes in RAW264 cells (C,D). Mag-AL/ATCOL complexes constructed at a mixing concentration ratio of 2:1 (Mag-AL:ATCOL) (C), and the complexes constructed using a fixed concentration of ATCOL (5 µg/mL) (D) were added to each well, and incubated for 30 min at 37 °C in the presence or absence of a magnetic field. Each value represents the mean ± SD (*n* = 4). **p ≤ .01, compared with the corresponding group with no magnetic field. ^††^*p*≤.01, compared with Mag-AL (ATCOL: 0 µg/mL) with a magnetic field.

We evaluated the effect of the concentration of ATCOL on the cellular association and/or uptake of Mag-AL/ATCOL complexes in RAW264 cells. As shown in Figure 2(A), the association of Mag-AL in RAW264 cells under a magnetic field was remarkably increased by mixing with ATCOL. The highest cellular association was observed with the complexes constructed using 5 µg/mL of ATCOL. However, the cellular association of the complexes was gradually diminished as the mixing concentration of ATCOL was increased above 10 µg/mL. Cytotoxicity was not observed with any of the complexes whether a magnetic field was involved or not (Figure 2(B) and Figure S1).

### Liposome concentration-dependent cellular association of Mag-AL/ATCOL complexes in RAW264 cells

From Figure 2(A), the highest cellular association of the Mag-AL/ATCOL complexes was obtained when the complexes were constructed by mixing Mag-AL and ATCOL at a concentration ratio of 2:1 (10 µg lipid/mL:5 µg/mL). Therefore, we assessed the liposome concentration-dependent cellular association of Mag-AL/ATCOL complexes constructed by mixing Mag-AL and ATCOL at a fixed ratio of 2:1. The cellular association and/or uptake of the complexes in RAW264 cells under a magnetic field showed linearity when the liposome concentration was up to 50 µg lipid/mL, but cellular association did not change between a liposome concentration of 50 µg lipid/mL and 100 µg lipid/mL (Figure 2(C)). We also investigated the cellular association of Mag-AL/ATCOL complexes constructed using a constant ATCOL concentration of 5 µg/mL. As shown in Figure 2(D), the cellular association of the complexes linearly increased in a Mag-AL concentration-dependent manner. Therefore, we decided that the optimal concentration of ATCOL for the construction of the Mag-AL/ATCOL complexes was 5 µg/mL, regardless of liposome concentration.

### Comparison of the cytotoxicity and cellular association of Mag-AL/ATCOL complexes with Mag-CL

**Figure 3.**
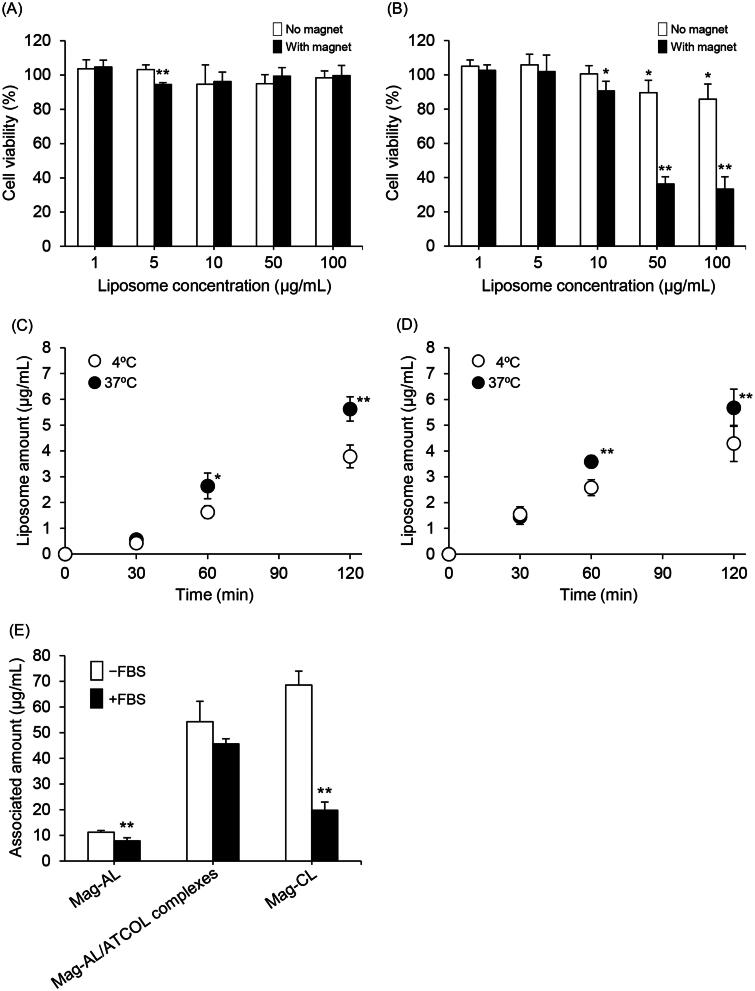
Comparison of the cytotoxicity and cellular association of Mag-AL/ATCOL complexes and Mag-CL. The cell viability of RAW264 cells incubated with Mag-AL/ATCOL complexes (A) and Mag-CL (B) for 30 min at 37 °C in the presence or absence of a magnetic field was assessed. Each value represents the mean + SD (*n* = 4). *p<.05; **p<.01, compared with the corresponding control group. Cellular association and/or uptake of the complexes (C) and Mag-CL (D) in RAW264 cells. One µg lipid/mL of the complexes or Mag-CL was added to each well, and incubated for 30–120 min at 4 °C or 37 °C in the presence of a magnetic field. Each value represents the mean ± SD (*n* = 4). *p<.05; **p<.01, compared with the corresponding group at 4 °C. (E) The effect of serum on the cellular association of Mag-AL/ATCOL complexes in RAW264 cells. A total of 10 µg lipid/mL of Mag-AL, Mag-AL/ATCOL complexes, and Mag-CL were incubated with 20% FBS for 30 min at 37 °C. Then, the liposomes were added to the well, and incubated for 30 min at 37 °C in the presence of a magnetic field. The complexes were constructed using a fixed concentration of ATCOL (5 µg/mL). **p<.01, compared with the corresponding non-FBS group.

We carried out a comparative cytotoxicity study between Mag-AL/ATCOL complexes and Mag-CL. As shown in Figure 3(A,B) and Figure S1, the Mag-AL/ATCOL complexes did not exhibit any cytotoxic effect against RAW264 cells, whereas liposome concentration-dependent cytotoxicity was observed with Mag-CL, particularly under a magnetic field.

We also compared the cellular association and uptake efficiency of the Mag-AL/ATCOL complexes and Mag-CL in RAW264 cells. The cellular association and/or uptake of the complexes at 37 °C increased in a time-dependent manner, and this profile was nearly identical to that of Mag-CL (Figure 3(C,D)). Moreover, we investigated the cellular association of the Mag-AL/ATCOL complexes and Mag-CL at 4 °C. Temperature greatly influences the interaction between nanoparticles and cells, and at 4 °C, energy-dependent endocytosis is significantly reduced (Fazlollahi et al., 2011; He et al., 2013). We observed that the cellular association of the complexes at 4 °C was significantly lower than that at 37 °C, and it was comparable to that of Mag-CL (Figure 3(C,D)).

We further evaluated the cellular association characteristics of the Mag-AL/ATCOL complexes in the presence of serum. As shown in Figure 3(E), the cellular association of Mag-CL was significantly decreased by approximately 70% in the presence of FBS, whereas that of the Mag-AL/ATCOL complexes were decreased by only 16%. This decrease was also smaller than that of Mag-AL (30%).

### Cellular association and cytotoxic effect of DXR-loaded Mag-AL/ATCOL complexes in CT-26 cells

**Figure 4.**
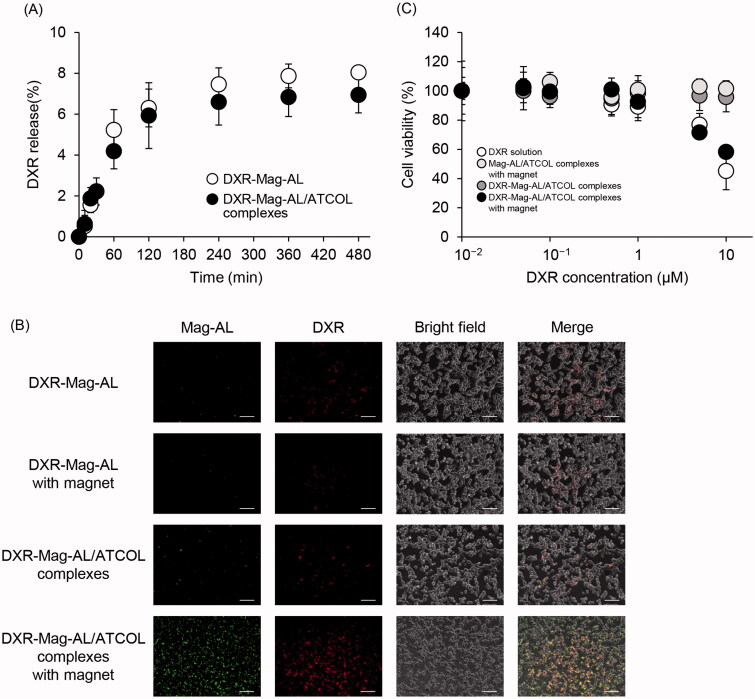
Cellular association and cytotoxicity of DXR-loaded Mag-AL/ATCOL complexes in CT-26 cells. (A) Release profile of DXR from Mag-AL/ATCOL complexes constructed using 5 µg/mL of ATCOL in PBS at 37 °C. (B) Representative fluorescent images of cellular association of DXR-loaded Mag-AL/ATCOL complexes in CT-26 cells. A total of 10 µg lipid/mL of the complexes was added to each well and incubated for 60 min at 37 °C in the presence or absence of a magnetic field. Scale bar, 10 µm. (C) Cell viability of CT-26 cells incubated with DXR-loaded Mag-AL/ATCOL complexes for 60 min at 37 °C in the presence or absence of a magnetic field. Each value represents the mean ± SD (*n* = 4).

To confirm the capacity of Mag-AL/ATCOL complexes as a drug carrier, DXR was loaded into Mag-AL by remote loading method. The particle size and *ζ*-potential of DXR-loaded Mag-AL/ATCOL complexes (particle size: 138.2 ± 4.5 nm, *ζ*-potential: −35.8 ± 3.3 mV) were similar with those of non-DXR loaded Mag-AL/ATCOL complexes. The loading efficiency of DXR into Mag-AL was more than 96% (96.3 ± 1.2%). Regardless of the mixing of ATCOL, approximately 7% of DXR was released from Mag-AL at 480 min following the incubation at 37 °C (Figure 4(A)), indicating that Mag-AL/ATCOL complexes are capable of stably incorporating DXR. We also evaluated the cellular association of DXR-loaded Mag-AL/ATCOL complexes in CT-26 cells. The remarked cellular association of both Mag-AL and DXR was observed when applying DXR-loaded Mag-AL/ATCOL complexes in the presence of a magnet (Figure 4(B)). In addition, the cellular associated pattern of DXR correlated well with that of Mag-AL. This result also suggests that DXR is stably encapsulated in Mag-AL. In investigating the cytotoxicity, DXR-loaded Mag-AL/ATCOL complexes with a magnet showed significant cytotoxicity, and the cytotoxicity was comparable with that of a DXR solution (Figure 4(C)).

### *In vivo* liver distribution of the Mag-AL/ATCOL complexes

**Figure 5.**
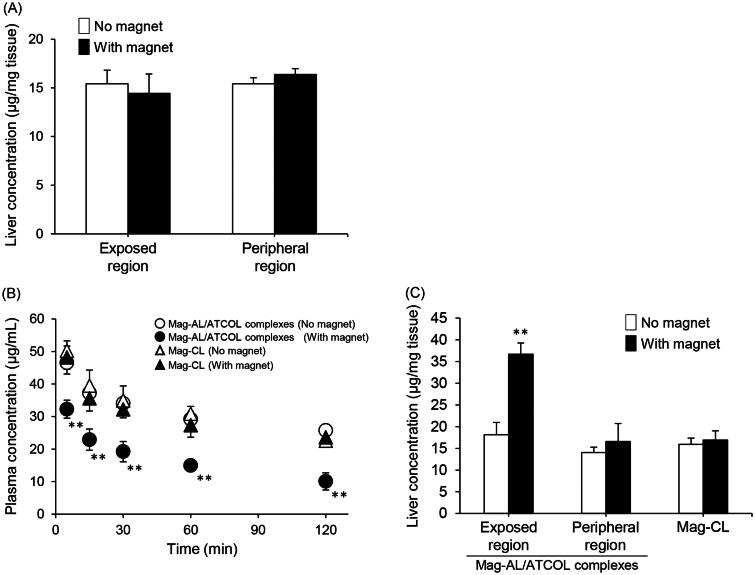
Plasma concentration–time profile and liver distribution of Mag-AL/ATCOL complexes after intravenous injection in rats. Liver accumulation of Mag-AL/ATCOL complexes constructed using 5 µg/mL of ATCOL (A), plasma concentration–time profile (B), and liver accumulation (C) of the complexes constructed using 10 µg/mL of ATCOL, and Mag-CL. The complexes or Mag-CL (400 µg of lipid) were intravenously injected into rats. Each value represents the mean + SD (*n* = 3). **p<.01, compared with the corresponding group with no magnetic field.

Figure 5 illustrates the plasma concentration–time profile and liver accumulation of the Mag-AL/ATCOL complexes after intravenous administration in rats. The liver accumulation of the complexes constructed using 5 µg/mL of ATCOL did not change with the presence or absence of a magnetic field (Figure 5(A)). When the complexes were constructed using 10 µg/mL of ATCOL, their plasma concentration was significantly decreased by the presence of a magnetic field (Figure 5(B)). Moreover, liver accumulation significantly increased at the magnetic field-exposed region (Figure 5(C)). In contrast, the blood circulation profile and liver accumulation of Mag-CL did not change in the presence or absence of a magnetic field (Figure 5(B,C)).

## Discussion

In the present study, we demonstrate that ATCOL greatly enhances the *in vitro* cellular association and uptake efficacy of Mag-AL in RAW264 cells without cytotoxicity, and Mag-AL/ATCOL complexes demonstrate selective liver accumulation under a magnetic field *in vivo*.

Initially, we attempted to optimize the mixing ratio of Mag-AL to ATCOL to prepare stable and monodispersed complexes. When Mag-AL was mixed with ATCOL, the particle size of the complexes increased in an ATCOL concentration-dependent manner (Table 1). It has been reported that the size of complexes consisting of ATCOL and negatively charged substances, such as proteins and oligonucleotides, changes depending on the concentration of the components (Hanai et al., 2006). Honma et al. (2001) have demonstrated that mixing pDNA with a low concentration of ATCOL results in nanoparticle complexes with a diameter of 100–200 nm, whereas mixing pDNA with a high concentration of ATCOL results in fibrous structures. Based on these observations, our present results suggest that Mag-AL form complexes with ATCOL, and the increase of the size of the complexes is attributable to a change in their structure from particulate to fibrous. The ATCOL concentration-dependent increase of the *ζ*-potential and TEM image also provide evidence that Mag-AL form complexes with ATCOL (Table 1, Figure 1(A)). In addition, we also visually confirmed that the complexes constructed using a higher ATCOL concentration (20–80 µg/mL) were significantly aggregated at 37 °C (Figure 1(B)). Since ATCOL has a tendency to form microfibrils under physiological conditions, such as at neutral pH and at 37 °C (Sano et al., 2003; Hanai et al., 2006), we assume that ATCOL at a concentration of more than 20 µg/mL forms a fiber-like structure at 37 °C in the mixture, resulting in the gelation of the complexes. Therefore, we decided that a lower concentration (1–10 µg/mL) of ATCOL is adequate to construct stable and monodispersed complexes with Mag-AL under physiological conditions.

In general, negatively charged nanoparticles undergo very little cell uptake because of the electrostatic repulsion between the nanoparticles and the anionic surface of the cells (Bannunah et al., 2014; Kurosaki et al., 2014; Kono et al., 2017). Our results clearly show that Mag-AL demonstrated significant cell association and cell uptake in RAW264 cells under a magnetic field when in complexes with ATCOL (Figure 2(A)). This is because ATCOL mediates the binding of Mag-AL to the cell surface via electrostatic interaction between the positively charged ATCOL and the anionic region on the cell membrane. However, the enhancing effect of ATCOL on the cellular association of Mag-AL was not so effective at higher ATCOL concentrations (20–80 µg/mL) compared with lower concentrations (1–10 µg/mL). It has been reported that large nanoparticles are not taken up by cells as efficiently as small nanoparticles (Bannunah et al., 2014; Langston Suen & Chau, 2014; Kono et al., 2017). Therefore, we assume that the cellular association and uptake of the Mag-AL/ATCOL complexes constructed using higher ATCOL concentrations is restricted because of their large size.

We observed that Mag-AL/ATCOL complexes constructed using a 5 µg/mL concentration of ATCOL showed liposome concentration-dependent cellular association (Figure 2(D)). However, the cellular association of complexes constructed at a fixed mixing concentration ratio of 2:1 (Mag-AL:ATCOL), reached a plateau at 50 µg lipid/mL of liposomes (Figure 2(C)). When the mixing concentration ratio is fixed, an increase in the Mag-AL concentration is accompanied by an increase in the ATCOL concentration. Therefore, Mag-AL/ATCOL complexes with a higher Mag-AL concentration would form a fibrous structure with a large diameter (Honma et al., 2001), resulting in the restriction of their cellular uptake. These results indicate that the absolute concentration of ATCOL, rather than the mixing ratio of ATCOL with Mag-AL, is a major determinant of the cellular association and uptake efficacy of Mag-AL/ATCOL complexes.

To achieve efficient drug delivery using liposomes, cationic lipids have been widely utilized because a cationic surface charge enables liposomes to associate with and internalize into cells efficiently (Dandamudi & Campbell, 2007; Shido et al., 2010; Bozzuto & Molinari, 2015; Kono et al., 2017). However, cationic lipids are known to cause severe toxic effects both *in vitro* and *in vivo* (Lv et al., 2006). We observed that Mag-CL exhibited a significant liposome concentration-dependent cytotoxicity under a magnetic field. However, the Mag-AL/ATCOL complexes did not have any cytotoxic effect against RAW264 cells (Figure 3(A,B) and Figure S1). This result is reasonable because both DSPG, which is a constitutive anionic lipid of Mag-AL, and ATCOL have been reported to show no cytotoxicity (Sano et al., 2003; Hanai et al., 2006; Kurosaki et al., 2014). In addition, we demonstrated that the cellular association and uptake of Mag-AL/ATCOL complexes in RAW264 cells under a magnetic field is comparable with that of Mag-CL. Furthermore, the time-dependent cellular association profile of the complexes at 4 °C, where low-temperature conditions cause a significant reduction in energy-dependent uptake, was also similar to that of Mag-CL (Figure 3(C,D)). These results indicate that the Mag-AL/ATCOL complexes are comparable with Mag-CL with regard to their capacity to bond to the cell surface and their cellular internalization efficiency. Minakuchi et al. have observed that ATCOL allows an increase in the cellular uptake of siRNA by forming complexes with siRNA (Minakuchi et al., 2004). Our results are in accordance with this report. Taken together, we reveal that ATCOL is a promising biomaterial for the improvement of cellular association and internalization of magnetic liposomes without cytotoxicity, and provides a superior alternative to conventional cationic lipids.

We also demonstrated that the Mag-AL/ATCOL complexes can stably encapsulate DXR and efficiently exhibit cytotoxicity against CT-26 cells under a magnetic field (Figure 4). These results indicate the potential of the Mag-AL/ATCOL complexes as an efficient magnetic-responsive drug carrier.

Considering the intravenous delivery of the Mag-AL/ATCOL complexes, it is important to evaluate the interaction of the complexes with serum. As shown in Figure 3(E), the cellular association of Mag-CL was markedly diminished by the presence of FBS. Since it has been reported that cationically charged polymers significantly interact with negatively charged serum proteins such as albumin (Lv et al., 2006), the reduction of the cellular association of Mag-CL may be caused by the adsorption of serum proteins on its surface. In contrast, the cellular association of the Mag-AL/ATCOL complexes was only decreased by 16%, with no significant difference between samples in the presence or absence of FBS. This result suggests that the complexes have a higher resistance to the adsorption of serum components or degradation in blood compared with Mag-CL. The reason why the Mag-AL/ATCOL complexes demonstrate such a resistance is unclear. However, since the complexes still had a negative *ζ*-potential when they were formed using a lower concentration (1–10 µg/mL) of ATCOL, we assume that the interaction of serum components with the complexes would be much weaker than that with Mag-CL, resulting in the higher stability of the complexes in serum. Finally, we evaluated the *in vivo* liver accumulation of the Mag-AL/ATCOL complexes after intravenous injection. We observed that the liver accumulation of the Mag-AL/ATCOL complexes in rats was significantly increased by the presence of a magnetic field, accompanied with a reduction in the plasma concentration (Figure 5(B,C)). This phenomenon was not observed with Mag-CL. Based on the result shown in Figure 3(E), this higher magnetic responsiveness of the Mag-AL/ATCOL complexes, in comparison with Mag-CL, may be attributed to their higher stability against serum components. In this experiment, the Mag-AL/ATCOL complexes were constructed using 10 µg/mL of ATCOL because the complexes constructed using 5 µg/mL of ATCOL did not respond to a magnetic field (Figure 5(A)). Since 500 µg/mL of Mag-AL was used to construct the complexes for the *in vivo* experiment, we consider that 5 µg/mL of ATCOL is insufficient to fully form the complexes with such a high concentration of Mag-AL.

In conclusion, we have succeeded in the construction of Mag-AL/ATCOL complexes as a potential MNP-based drug carrier. Our present observations make a valuable contribution towards the development of an anionic nanoparticle-based safe and efficient magnetic drug delivery system.

## Supplementary Material

Yusuke_Kono_et_al_supplemental_content.zip
