# Supplementary figures and images for: Development of magnetic anionic liposome/atelocollagen complexes for efficient magnetic drug targeting
Source: Drug Deliv. 2017 Nov 15;24(1):1740–9. doi: 10.1080/10717544.2017.1402219 (PMC8241088; doi:10.1080/10717544.2017.1402219)

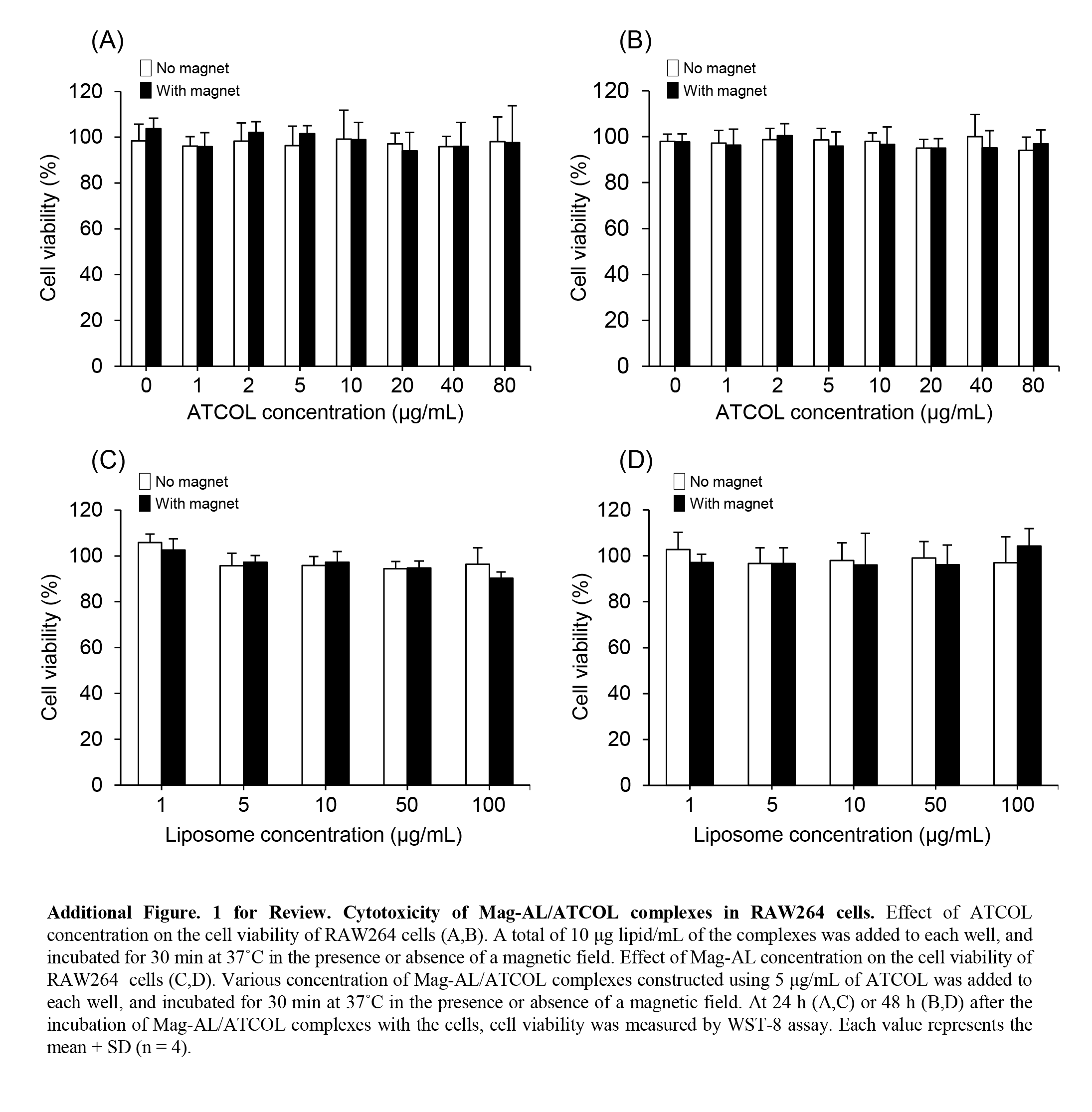

Supplement: Yusuke_Kono_et_al_supplemental_content.zip [file IDRD_A_1402219_SM9377.zip › Yusuke Kono et al supplemental content.tif]
